# Knowledge, attitude, and practice of pharmacy and medical students regarding self-medication, a study in Zabol University of Medical Sciences; Sistan and Baluchestan province in south-east of Iran

**DOI:** 10.1186/s12909-020-02374-0

**Published:** 2021-01-14

**Authors:** Mahmoud Hashemzaei, Mahdi Afshari, Zahra Koohkan, Ali Bazi, Ramin Rezaee, Kaveh Tabrizian

**Affiliations:** 1 Department of Pharmacology and Toxicology, Faculty of Pharmacy, Zabol University of Medical Sciences, Zabol, Iran; 2 Toxicology and Addiction Research Center, Zabol University of Medical Sciences, Zabol, Iran; 3 Department of Community Medicine, Faculty of Medicine, Zabol University of Medical Sciences, Zabol, Iran; 4 Students Research Committee, Faculty of Pharmacy, Zabol University of Medical Sciences, Zabol, Iran; 5 Faculty of Allied Medical Sciences, Zabol University of Medical Sciences, Zabol, Iran; 6 Clinical Research Unit, Faculty of Medicine, Mashhad University of Medical Sciences, Mashhad, Iran; 7 Neurogenic Inflammation Research Center, Mashhad University of Medical Sciences, Mashhad, Iran

**Keywords:** Self-care, Prevalence, Medical students, Drugs, Antibiotic resistance

## Abstract

**Background:**

Self-medication is defined as using medicinal products to treat the disorders or symptoms diagnosed by oneself. Although informed self-medication is one of the ways to reduce health care costs, inappropriate self-treatment can pose various risks including drug side effects, recurrence of symptoms, drug resistance, etc. The purpose of this study was to investigate the knowledge, attitude, and practice of pharmacy and medical students toward self-medication.

**Methods:**

This study was conducted in Zabol University of Medical Sciences in 2018. Overall, 170 pharmacy and medical students were included. A three-part researcher-made questionnaire was designed to address the students’ knowledge, attitude, and practice. Statistical analysis was performed in SPSS 25 software.

**Results:**

According to the results, 97 (57.1%) students had carried out self-medication within the past 6 months. Overall, the students self-medicated on average 4.2 ± 2.9 times per year. Self-medication was more common in male students (65.4%, *P* = 0.043). Cold was the most common ailment treated with self-medication (93.2%), and antibiotics (74.4%) were the most commonly used drugs. The primary information sources used by the students were their previous prescriptions (47.4%). Pharmacy students had a higher level of drug information (*P* < 0.001). There was a statistically significant association between the level of drug information and the tendency for self-medication (*P* = 0.005). Disease recurrence was the most common negative complication of self-medication.

**Conclusion:**

There is a need to educate pharmacy and medical students regarding self-medication and its side effects. The high prevalence of self-medication and the overuse of antibiotics can pose a significant risk of drug resistance.

**Supplementary Information:**

The online version contains supplementary material available at 10.1186/s12909-020-02374-0.

## Background

Self-medication is defined as using medicinal products to self-medicate disorders or their symptoms. Overusing the medications prescribed by a physician for oneself or other family members (especially when it comes to children or the elderly) also falls into the definition of self-medication [1]. The prescription of medicines for oneself without having specialists’ advice can cause many side effects including drug resistance and complications, as well as prolonged disease course [2].

Responsible self-medication includes using over the counter (OTC) and relatively low-risk drugs to treat self-diagnosed disorders or symptoms [3], which can prevent mild illnesses and thereby reduce health care financial costs. In order to safely and effectively use a prescription drug, the consumer must accurately identify symptoms, ascertain therapeutic goals, and use appropriate drugs, dosage, and therapy durations. Furthermore, medical history, contraindications, concomitant co-morbidities, potential adverse effects, and finally response to treatment should be precisely monitored.

The prevalence of self-medication widely varies in different countries. For example, in Spain [4], Chile [5], Vietnam [6], China [7], and India [8], the prevalence of self-medication have been 12.7, 75%, 40–60, 32, and 71%, respectively. In Iran, the estimated per capita drug consumption is relatively high, from which self-medication shares a substantial part [9]. Factors such as gender, income, and drug information can affect the tendency toward self-medication [10].

The prevalence of self-medication among different social groups in Iran ranges from 35 to 90% [11, 12]. Pain killers, eye drops, and antibiotics bear the largest share of the drugs used by Iranians for self-medication [13, 14]. The most important factors encouraging self-medication in Iran and the world are suffering from mild symptoms, having prior drug prescriptions or insurance problems, lack of awareness, ease access to drugs, and favorable cultural and socio-economic status [12, 15]. The most important diseases self-medicated in Iran are respiratory diseases, cold, and headaches [16]. The studies conducted in different parts of Iran have revealed a higher prevalence of self-medication among students than the general population [11, 17, 18].

Pharmacists and physicians play key roles in providing helpful recommendations on proper and safe use of pharmaceutical products. Therefore, the purpose of this study was to investigate knowledge, attitude, and practice toward self-medication in pharmacy and medical students.

## Methods

The present descriptive cross-sectional study was conducted on the pharmacy and medical students studying at Zabol University of Medical Sciences, Sistan and Baluchestan province in the south-east of Iran in 2018. The university, which is supported by the government, was established in 2002 in order to provide health services and education to the people of the north region of Sistan and Baluchestan province. The organization currently offers health education to students in 25 study fields.

### Sample size

Considering the good knowledge and attitude rates of 50, 95% confidence interval, and the maximum error rate of 7%, sample size was determined as 170 using the following formula:

$$ \mathrm{n}=\frac{{\mathrm{z}}^2\ast p\left(1-p\right)}{{\mathrm{d}}^2} $$

Pharmacy and medical students sequentially entered into the study. The sampling method was based on multi-stage random sampling so that medical and pharmacy schools as well as each entry-year were considered as stratums, and within the stratums, students were randomly selected. For interns who did not attend classes, the researcher referred to their internship hospitals (Amir-Al-Momenin and Imam Khomeini hospitals of Zabol city) where the students were initially selected by the stratified method and then simple random sampling to achieve the required sample size.

### Data collection

A researcher-made questionnaire (supplementary file 1) was used to collect the data. The questions were organized in three parts to assess knowledge, attitude, and practice. For knowledge, the students were initially asked if they could correctly name three OTC drugs, and then they were given six statements and asked to determine whether these statements were true or false. The true answer was given 1 score, and the false and “don’t know” responses were assigned with the scores of − 1 and 0, respectively. Total knowledge score was categorized as good (≥4), average (1–3), or poor (0 or lower) based on the method described by Isacson and Bingeforse [19]. For attitude, the students were asked to rate their agreement or disagreement toward multiple propositions about self-medication. For practice, questions were asked about the types of drugs and ailments, as well as reasons for and the negative outcomes of self-medication.

### Validity and reliability

The content validity of the questionnaire was ensured with the help of pharmacists, physicians, and epidemiologists by incorporating the necessary corrections suggested by them. The reliability of the questionnaire was approved after being completed by 30 students at two occasions. These students were chosen from medical and pharmacy faculties and different educational years. They were asked to gather in a classroom at a specific time not to interfere with their classes or other educational activities. They were explained about the aim of the study and asked to fill the questionnaire. The same procedure was followed again after 2 weeks, and finally the Cronbach’s alpha coefficient was calculated. To reach an acceptable coefficient, some modifications were performed on four questions of the attitude domain, and one question was also omitted in this section (Cronbach’s alpha coefficient = 0.44). The researcher attended the students at the time of completing the questionnaire to resolve any ambiguity.

### Statistical analysis

SPSS 25 software was used to analyze the data. Quantitative and qualitative variables were described by mean ± standard deviation and percentage, respectively. The distributions of the variables were assessed by the Kolmogorov–Smirnov test. Quantitative variables were compared by independent samples student t-test (for two-group comparisons) and one-way ANOVA (for comparisons among three or more groups). Between-group comparisons of qualitative variables were performed by the Chi-square test. *P* value < 0.05 was considered as statistically significant.

## Results

### Demographic information

In this study, 170 students were interviewed from whom 78 (45.9%) were males and 92 (54.1%) were females, and all of them completed the survey. The mean age of the students was 21.92 ± 1.8 years. The youngest and oldest participants aged 18 and 29 years old, respectively. Of the participants, 105 (61.8%) and 65 (38.2%) were medical and pharmacy students, respectively. In terms of the year of enrollment, most participants (22.4%) were senior (3 years or higher). Demographic information has been shown in Table 1.

**Table 1:** Demographic features of 170 medical and pharmacy students

Variables		N	%
Gender	Male	78	45.9
	Female	92	54.1
Field of study	Medicine	105	61.8
	Pharmacy	65	38.2
Residency	Dorm	129	75.5
	With family	36	21.2
	Leased	5	2.9
Chronic disease	Yes	18	10.6
	No	152	89.4

### History of self-medication

Out of 170 students, 97 (57.1%) self-medicated within past 6 months. A significant association was found between self-medication and gender (*p* = 0.043), but not the field of study, residency, and history of chronic diseases (Table 2).

**Table 2:** The association of self-mediation within the past 6 months with demographic variables in 170 medical and pharmacy students

Variables		Self-treatment		*P*
		Yes	No	
Gender	Male	51 (65.4)	27 (34.6)	0.043
	Female	46 (50)	46 (50)	
Field of study	Medicine	60 (57.1)	45 (42.9)	0.978
	Pharmacy	37 (56.9)	28 (43.1)	
Chronic diseases	Yes	12 (66.7)	6 (33.3)	0.456
	No	85 (55.9)	67 (44.1)	

### Students’ knowledge and attitudes toward self-medication

When the participants were asked to name three drugs that can be obtained without a prescription (i.e. OTC drugs), 12.9% were able to provide completely correct answers (Table 3). In terms of the knowledge score, there was no difference in the level of knowledge comparing students with or without a history of self-medication (*P* = 0.480). The level of knowledge was significantly associated with the field of study (*p* < 0.001), year of entrance (*p* = 0.002), and the history of self-medication (*p* = 0.005) (Table 4). The students’ attitudes toward the statements about self-medication have been presented in Table 5.

**Table 3:** The level of awareness of medical and pharmacy students from OTC drugs

Frequency of correct answers	N	%
3/3	22	12.9
2/3	45	26.5
1/3	44	25.9
0/3	8	4.7
No knowledge	51	30
Total	170	100

**Table 4:** The level of knowledge among medical and pharmacy students regarding self-medication

Variables		Awareness			*P*
		Good	Moderate	Poor	
Gender	Male	19 (24.4)	39 (50)	20 (25.6)	0.228
	Female	32 (34.8)	35 (38)	25 (27.2)	
Field	Medicine	16 (15.2)	52 (49.5)	37 (35.2)	< 0.001
	Pharmacy	35 (53.8)	22 (33.8)	18 (12.3)	
Year of education	First year	2 (4.5)	25 (56.8)	17 (38.6)	0.002
	Second and more	49 (38.9)	49 (38.9)	28 (22.2)	
Self-treatment	Yes	38 (39.2)	40 (41.2)	19 (19.6)	0.005
	No	13 (17.8)	34 (46.6)	26 (35.6)

**Table 5:** Medical and pharmacy students’ attitudes regarding self-medication

Statements	Attitude				
	Completely agree	Agree	No idea	Disagree	Completely disagree
Self-treatment is part of self-care	19 (11.2)	51 (30)	41 (24.1)	47 (27.6)	12 (7.1)
Would you like to start or continue your therapy?	22 (12.9)	60 (35.3)	40 (23.5)	36 (21.2)	12 (7.1)
Do you recommend self-treatment to others?	10 (5.9)	24 (14.1)	45 (26.5)	57 (33.5)	34 (20)
Should drug release be free?	9 (5.3)	14 (8.2)	35 (20.6)	67 (39.4)	45 (26.5)
Need No Training on the Disadvantages of Self-Treatment?	11 (6.5)	13 (7.6)	31 (18.2)	53 (31.2)	62 (36.5)
There is no need to try to simplify access to health care facilities	9 (5.3)	16 (9.4)	30 (17.6)	51 (30)	64 (37.6)

### Students’ performance regarding self-medication

**Fig. 1:**
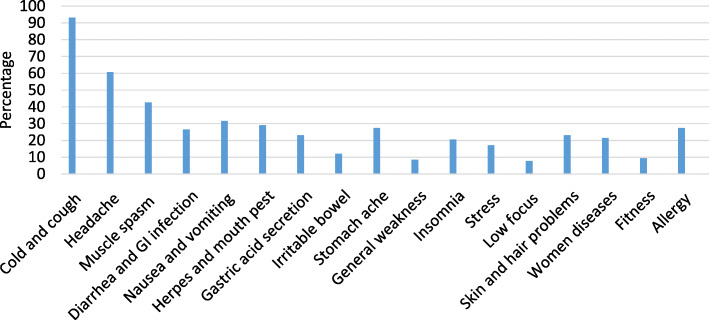
Most common diseases treated with self-medication among medical and pharmacy students

Overall, the students self-medicated on average 4.2 ± 2.9 times per year. Modern medicine (allopathy) with 69.2% was the most frequently used method in comparison with traditional Islamic medicine (29.9%) and other types such as Indian medicine, homeopathy, etc. (10.3%). Cold, headache, and muscle pain were the most prevalent ailments treated by self-medication with the prevalence of 93.2, 60.7, and 42.7%, respectively (Fig. 1).

### Drugs used for self-medication

**Fig. 2:**
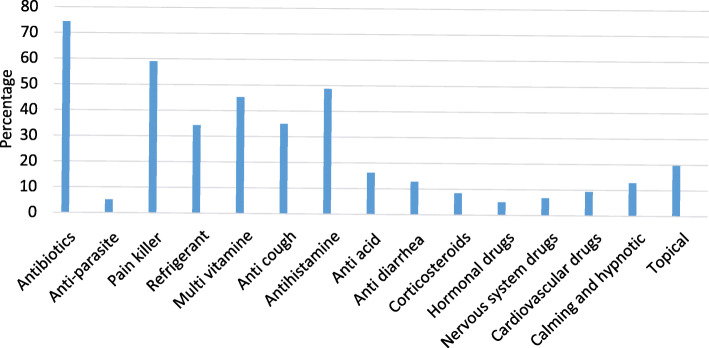
Most common drugs used by medical and pharmacy students for self-medication

Antibiotics were the most common drugs used for self-medication with a prevalence of 74.4%. Painkillers (59%) and antihistamine (48.7%) were the next most commonly used drugs (Fig. 2).

### Reasons of self-medication

**Fig. 3:**
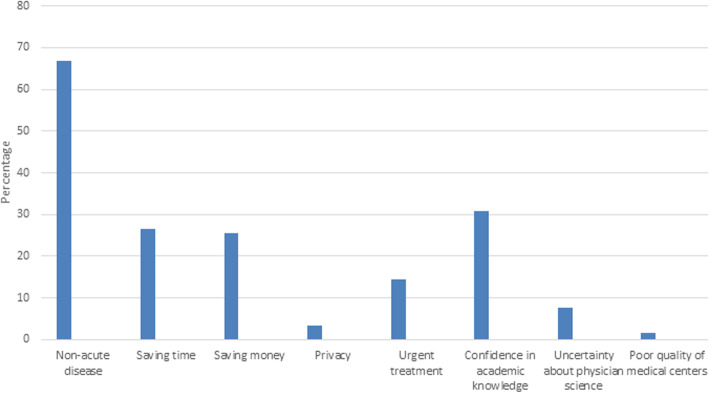
Common reasons for self-medication among Iranian pharmacy and medical students

The most common incentives encouraging the students to self-medicate were the illness being mild and reliance on one self’s academic knowledge (Fig. 3).

### Information resources

**Fig. 4:**
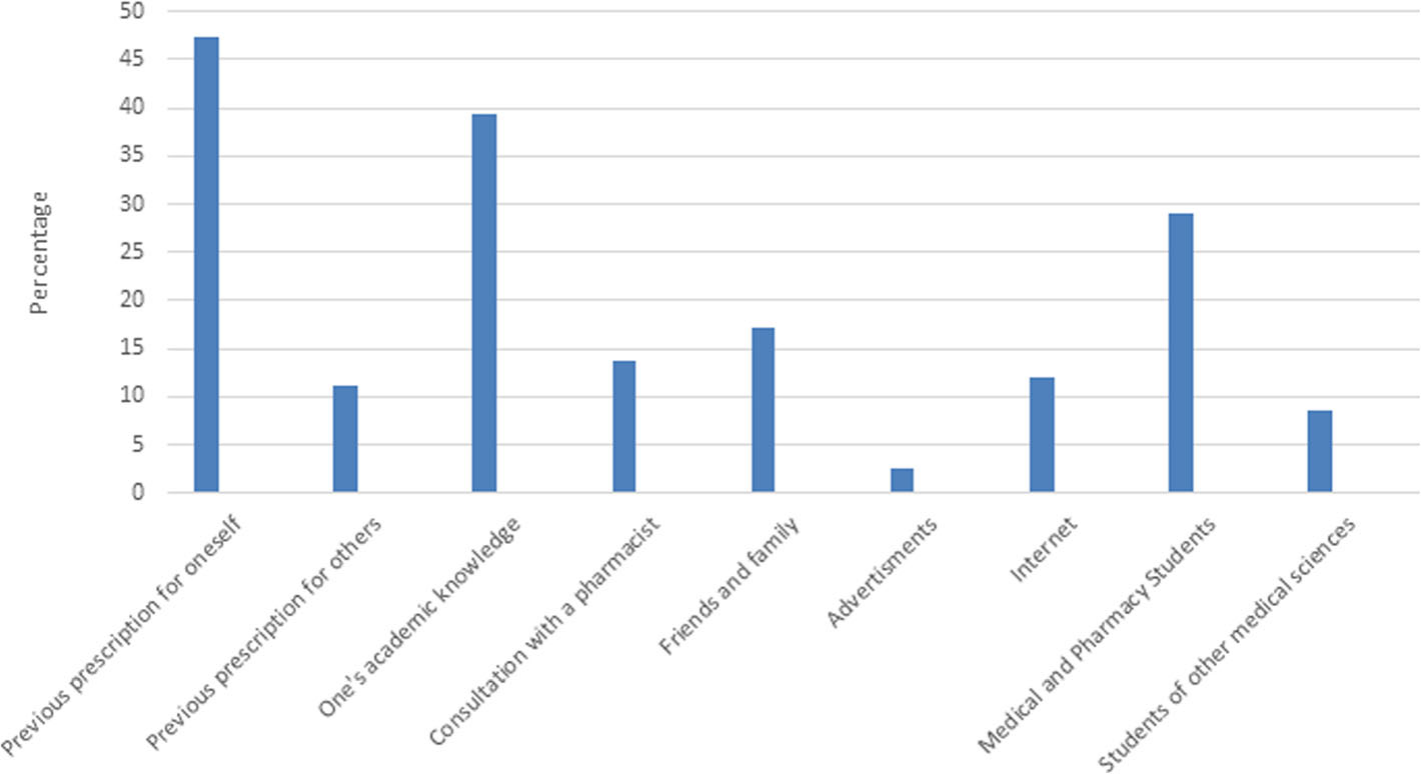
Information sources used by pharmacy and medical students to self-medicate

Information resources used by the students to self-medicate have been shown in Fig. 4. Most students (47.4%) used their previous prescriptions, and 39.3% used their own academic knowledge.

### Negative impacts of self-medication

**Fig. 5:**
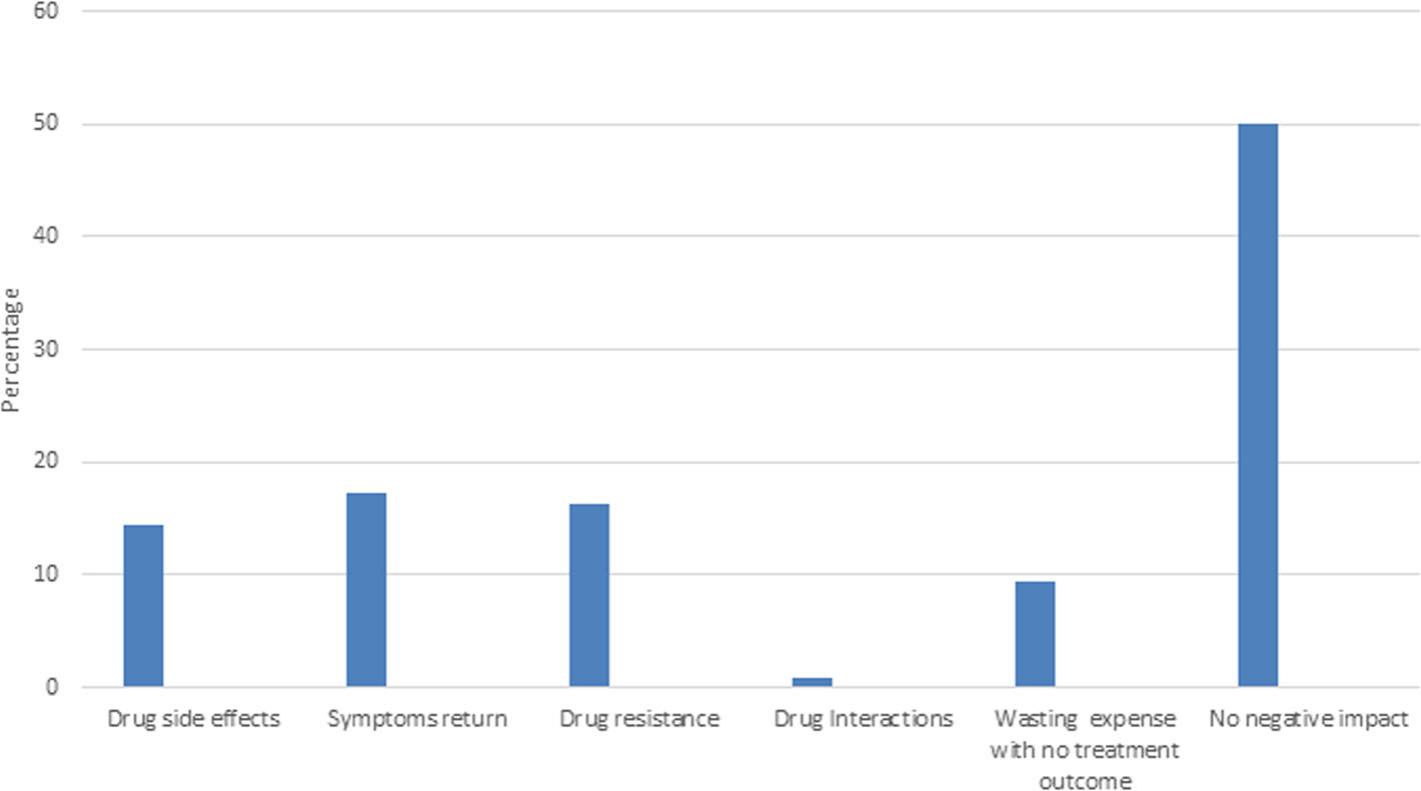
Negative impacts of self-medication among Iranian pharmacy and medical students

Figure 5 shows that disease recurrence was the most common negative complication of self-medication. On the other hand, 50% of the students reported no negative impacts.

## Discussion

Our study showed that 57.1% of the pharmacy and medical students of Zabol University of Medical Sciences who participated in the study had at least one episode of self-medication during the past 6 months. The prevalence of self-medication in Iran and other countries highly varies among different demographic groups. For example, a similar study on medical and pharmacy students in Ethiopia reported a prevalence of 38.5% [20]. Also, 44.8% of Bahraini [21], 78.6% of Indian [22], and 55.2% of Egyptian [23] medical students reported episodes of self-medication. In another study, 98% of Palestinian students [24] reported self-medication. Among studies in European countries, two that were performed on Slovenian [25] and Serbian [26] students reported the frequencies of 92.3 and 79.9%, respectively. A study on a Spanish adult population also reported 45% prevalence for self-medication to treat cold [27]. A study on German adolescents described the prevalence of 8% for self-medication [28]. In another study on patients with gastro-esophageal reflux in France, self-medication was reported by 17% of the participants [29]. Overall, the results of the present study showed that the prevalence of self-medication was much higher than that of developed but similar to the values reported in developing countries. It has been shown that the prevalence of self-medication is generally higher in developing compared with developed countries [30]. This can be due to differences in the levels of welfare and income per capita reflecting the higher ability for paying for health care costs, as well as the better quality of health care services and more efficient drug supply monitoring programs in developed countries [31].

Among the studies conducted in other parts of Iran, self-medication was reported in 91% of students in Kerman [12], 83% in Yazd [32], and 80% in Ardabil [33]. A review conducted in 2015 by Azami et al. reported the frequencies of 53 and 67% for self-medication in the general population and students in Iran, respectively [34]. These were close to the prevalence reported in the present study. Self-medication seems to be higher among students than the general population. This can be due to a variety of reasons such as students’ higher pharmaceutical and clinical knowledge, their better access to the Internet and the media advertising pharmaceutical products, and the cost-effectiveness of self-medication for them [35]. Nevertheless, different populations, research design, and data analysis methods make it difficult to compare the prevalence of self-medication among different societies.

Regarding knowledge, only 12.9% of our students were able to correctly name three OTC drugs. The ratio of students who could recall 2 or more OTC drugs was 39.4%, and the rest of them knew either one or none of OTC drugs. Overall, 16 and 35% of our medical and pharmacy students achieved good scores regarding drug information. The ratio of pharmacy students who had good knowledge on this issue was significantly higher in comparison with medical students (*P* < 0.001). Furthermore, senior students (two or more years of education) had significantly higher knowledge than first-year students (*P* = 0.002). This observation probably reflects differences in educational courses and curriculums of these fields. Similarly, in a study on pharmacy students in Addis Ababa, Ethiopia, 47.3% of students did not know any OTC drug. In this study, the participants’ knowledge scores were categorized using the method presented by Isacson and Bingforse [19]. Overall, 26.5% of the students had poor scores while 43.5 and 30% attained average and good scores, respectively. In line, in a similar study on Palestinian students using the same grading method, it was reported that one-third of the students had poor scores while others acquired average and good scores [24]. This observation probably reflects the higher number of drug courses of pharmacy students and their deeper integration with pharmaceutics. Also, senior students (two-year and higher) had higher levels of drug information than juniors (one-year), indicating the impact of educational courses on the students’ drug knowledge. In general, medical and pharmacy students seem to need more effective educations in this area.

In the present study, the only variable that had a significant impact on the attitude was the field of study so that pharmacy students had more negative attitudes than medical students. This may be related to the higher drug knowledge of pharmacy students. In fact, students with higher drug information were more likely to self-medicate; however, there was no statistically significant difference in the prevalence of self-medication between the medical and pharmacy students. The presence or absence of chronic diseases also had no significant impact on the rate of self-medication among the students. In a review study, Isacson et al. described an association between drug knowledge and a positive attitude toward self-medication [19]. A study by James et al. in 2005 also noted that higher levels of medical and pharmaceutical knowledge make people more cautious about taking and recommending medications [21]. In our study, male students were more likely to self-medicate than females. This observation was different from that of two other studies in which females had self-medicated more commonly than males [22, 23]. In a number of studies; however, there were no significant differences between males and females in this regard [21, 25]. A study on university students reported that a history of self-medication was significantly associated with age, gender, and the year and field of study [36]. Of other factors associated with self-medication have been low medical information and histories of alcohol use and tobacco smoking [37]. In fact, many individuals who perpetrate self-medication actually perceive this as a safe phenomenon [38]. Overall, many variables seem to affect the tendency for self-medication, and the knowledge seems to be a predominant factor.

Antibiotics (74.4%) and painkillers (60%) were the most commonly used drugs for self-medication among our students. In other studies, NSAIDs [37, 39], antibiotics [36, 39, 40], pain killers [36, 37], as well as anti-flu [37] and anti-malarial [36] drugs have been the most common pharmaceutics used for self-medication. In two studies on university students, paracetamol [36] and amoxicillin [40] were reported as the most common drugs used for self-medication. In a study in Pakistan, OTC drugs comprised the most common (98.3%) pharmaceuticals used by medical undergraduate students [41]. In another study on first-year medical students in Bahrain, 6% of the participants used antibiotics for self-medication [21]. Also, this rate was reported to be 17.2% in Ethiopia [42], 38.9% in Serbia [26], 19.9% in Palestine [24], and 34% in India [43]. Using antibiotics for self-medication is much less common in developing than developed countries [44]. In a study by Aljinovic et al. in Croatia, the researchers found that using antibiotics for self-medication was higher among healthcare workers than the general population [45], supporting our observation in this study. Overall, the rate of using antibiotics for self-medication observed here was similar to most of the studies conducted in Iran and higher than that reported in other parts of the world. According to this, the risk of antibiotic resistance may be a serious threat to our society in future.

Regarding our students’ performance, cough and cold along with headache were the most common self-treated ailments. In a study on 360 Iranian women, fever, fatigue, and anxiety were the most common ailments managed by self-medication [38]. Cold, fever, and cough were the main reasons of self-medication in another study on 570 university students in Rwanda [40]. Likewise, our results were consistent with the findings of most similar studies [23, 42, 46]. The most important reason for self-medication was noted to be the non-acute nature of the disease. This was consistent with a previous report [47]. A non-severe or transient disease was also the most common reason for self-medication (45–46%), according to two studies in China [48] and Brazil [39]. In the Chinese report, 23% of participants were reluctant to see a doctor over a relatively mild condition, and 12% noted that they did not have enough time to see a physician. In a study in Rwanda, not having a serious illness was also the main pretest of university students for self-medication [40]. Financial shortcomings and insurance problems have been reported as other reasons for self-medication [38, 48]. In a study in Brazil, nursing students justified self-medication by noting that they were unaware of its potential side-effects and complications [39]. In another report, bad behavior of health care providers, the clinic being too far, and low efficiency of prescribed drugs were noted as excuses for self-medication among university students [36]. Another reason encouraging people to self-medicate may be storing drugs at homes. In one study, 98.9% of Iranian women who self-medicated had a pile of stored drugs at their homes [38]. Also, in the recent report, having a history of the same disease was declared as another reason for self-medication [38].

An important point in our study was the students’ low tendencies to consult with a pharmacist as a reliable and accessible source to ensure safe self-medication. Only 13.7% of the students mentioned that they consulted with a pharmacist before self-medication. This may reflect the fact that these students were aware of the risks associated with self-medication, but at the same time, they believed that they were equipped with sufficient knowledge to remain safe. Unwanted interactions between drugs, drug-dependency, and choosing inappropriate drugs because of an incorrect diagnosis are among the factors threatening the health of those who self-medicate [49]. The importance of the health side effects of self-medication becomes more pronounced knowing that people may not follow the correct instructions of drug usage, consume excessive doses, or simultaneously consume prescription and non-prescription drugs, which can exaggerate the risk of health-threatening complications [37]. So, medical and pharmacy students seem to need more education about the risks of irresponsible self-medication.

### Strengths and limitations

This was a cross-sectional study, so it is advisable to investigate the trend of students’ tendency toward self-medication in prospective studies and evaluate the factors that may influence their attitudes and practice toward self-medication. As our study relied on the ability of students to recall using drugs, a bias is inevitable due to this issue. To manage this sort of bias, we restricted the time period to prior 6 months. Furthermore, the first-year students included in this study may have created some heterogeneity in the field of knowledge. On the other hand, a high response rate (100%) was one of the strengths of this study.

## Conclusions

Responsible self-medication is one of the main strategies to reduce health care costs, so it seems that the role of pharmacists and pharmacy students is particularly important in this regard. Therefore, pharmacists should be regarded as major contributors to the public health care system, and medical and pharmacy students, as future health professionals, should be more educated about good pharmacy practice and responsible self-medication.

## Supplementary Information

**Additional file 1.** Supplement. This is the questionnaire used in this study.

## Data Availability

The datasets used and/or analysed during the current study are available from the corresponding author on reasonable request.

## Abbreviations

OTC: Over the counter
