# Supplementary material for: Knowledge, attitude, and practice of pharmacy and medical students regarding self-medication, a study in Zabol University of Medical Sciences; Sistan and Baluchestan province in south-east of Iran
Source: BMC Med Educ. 2021 Jan 14;21:49. doi: 10.1186/s12909-020-02374-0 (PMC7807440; doi:10.1186/s12909-020-02374-0)
Supplement: Supplementary file 1 — Additional file 1. Supplement. This is the questionnaire used in this study. [file 12909_2020_2374_MOESM1_ESM.docx]

**Dear student, this questionnaire is just a part of a research project. Your answers will be confidential and use for research purpose only.**

**Part A)**

**Please name three OTC drugs.**

**Part B)**

**Please mark what is true for you in front of each statement.**

| **Statements** | Fully agree | Agree | No idea | Disagree | Fully Disagree |
| --- | --- | --- | --- | --- | --- |
| **Self-treatment is part of self-care** |  |  |  |  |  |
| **I would like to start or continue your therapy?** |  |  |  |  |  |
| **Do you recommend self-treatment to others?** |  |  |  |  |  |
| **Should drug release be free?** |  |  |  |  |  |
| **Need No Training on the Disadvantages of Self-Treatment?** |  |  |  |  |  |
| **There is no need to try to simplify access to health care facilities.** |  |  |  |  |  |

**Part C)**

**Please respond to following questions**

**Q1: How many times do you usually self-medicate per year?**

□1 □2 □3 □>3

**Q2: What type of medicine do you use when self-medicate?**

□ Modern □Islamic-traditional □Other (Indian, etc.)

**Q3: For what diseases do you usually use self-mediation?**

□ Cold and cough □Headache □Sore muscle □Diarrhea and intestinal problems □Nausea □ Herpes simplex and oral plagues □Heartburn □IBD □Abdominal pain □ Fatigue □Insomnia □Stress □Focus problems □Cutaneous and skin problems □Women diseases □Fitness □Allergies □Other (please specify)

**Q4: What drugs do you usually used for self-medication?**

□Antibiotics □Ant-parasites □Pain killers □Antipyretic □Multivitamins □Anti-cough □Anti-histamine □Anti-acid □Anti-diarrhea □Corticosteroids □Hormonal drugs □Neuronal drugs □Cardiovascular drugs □Pacifiers and sleeping pill □Topical medicine □other (please name)

**Q5: For what reason do you prefer to self-medicate instead of going to visit a doctor?**

□The disease not being serious □Saving time □Saving money □Privacy □ Urgent need to use the drug □Confidence in my own knowledge □Not believing in the physician □Low quality of health care services □Other (please specify)

**Q6: What information source do usually you use for self-medication?**

□Prior prescriptions of my own □Previous prescriptions of others □My own academic knowledge □Counseling with the pharmacist □Friends and relatives □Advertisements □Internet □Other medical and pharmaceutical students □ Non-medical students □Other (please specify)

**Have you ever experienced a negative side effect after self-medication?**

□ No

□ Yes

**If yes, please specify:**

□Drugs side effects □Disease recurrence □Resistance to drug □Drug interactions □ No therapeutic effects □Other (please name)
